# Supplementary material for: How oscillating aerodynamic forces explain the timbre of the hummingbird’s hum and other animals in flapping flight
Source: eLife. 2021 Mar 16;10:e63107. doi: 10.7554/eLife.63107 (PMC8055270; doi:10.7554/eLife.63107)
Supplement: Supplementary file 1. — To obtain frequency resolution ≤ 2 Hz, we selected feeding flights of 0.5 s or longer. [file elife-63107-supp1.docx]

| Hummingbird | #1 | #2 | #3 | #4 | #5 | #6 |
| --- | --- | --- | --- | --- | --- | --- |
| # feedings recorded | 4 | 3 | 4 | 10 | 4 | 3 |
| # feedings > 0.5 s | 3 | 1 | 2 | 6 | 3 | 3 |
