# Supplementary material for: How oscillating aerodynamic forces explain the timbre of the hummingbird’s hum and other animals in flapping flight
Source: eLife. 2021 Mar 16;10:e63107. doi: 10.7554/eLife.63107 (PMC8055270; doi:10.7554/eLife.63107)
Supplement: Supplementary file 2. — To investigate how well hummingbird flapping wing hum can be approximated with a single acoustic source per wing, we created a distributed oscillating source model with ten equally spaced elements along each wing. The force distribution was adapted from a high-fidelity model by Ingersoll and Lentink, 2018 for the same hummingbird species. There is close agreement in magnitude for the first ten harmonics of the single and ten source model. [file elife-63107-supp2.docx]

| Harmonic | 1^st^ | 2^nd^ | 3^rd^ | 4^th^ | 5^th^ | 6^th^ | 7^th^ | 8^th^ | 9^th^ | 10^th^ |
| --- | --- | --- | --- | --- | --- | --- | --- | --- | --- | --- |
| 10 Element Model [dB] | 55.1 | 56.9 | 48.3 | 40.5 | 33.4 | 30.9 | 32.9 | 28.1 | 31.5 | 23.1 |
| Equivalent Point Source [dB] | 55.3 | 57.0 | 48.4 | 40.5 | 33.4 | 30.9 | 32.9 | 28.1 | 31.5 | 23.1 |
