# Supplementary material for: How oscillating aerodynamic forces explain the timbre of the hummingbird’s hum and other animals in flapping flight
Source: eLife. 2021 Mar 16;10:e63107. doi: 10.7554/eLife.63107 (PMC8055270; doi:10.7554/eLife.63107)
Supplement: Supplementary file 3. — There is reasonable agreement in magnitude for the first four harmonics. [file elife-63107-supp3.docx]

| Harmonic | 1^st^ | 2^nd^ | 3^rd^ | 4^th^ | 5^th^ | 6^th^ | 7^th^ | 8^th^ | 9^th^ | 10^th^ |
| --- | --- | --- | --- | --- | --- | --- | --- | --- | --- | --- |
| Full Model [dB] | 55.3 | 57.0 | 48.4 | 40.5 | 33.4 | 30.9 | 32.9 | 28.1 | 31.5 | 23.1 |
| Simplified Model [dB] | 56.3 | 58.0 | 41.8 | 40.4 | 27.3 | 25.0 | 12.1 | -2.9 | -6.2 | -8.1 |
