# Supplementary material for: How oscillating aerodynamic forces explain the timbre of the hummingbird’s hum and other animals in flapping flight
Source: eLife. 2021 Mar 16;10:e63107. doi: 10.7554/eLife.63107 (PMC8055270; doi:10.7554/eLife.63107)
Supplement: Supplementary file 5. — Culex quinquefasciatus was adapted from Bomphrey et al., 2017. Drosophila hydei mass was adapted from Greenewalt, 1962, while the other parameters were adapted from Muijres et al., 2014. Manduca sexta parameters were adapted from Zheng et al., 2013. Calypte anna values were obtained from the present experiment. Forpus coelestis values were adapted from Chin and Lentink, 2017. To simplify the comparison between the five paradigm animals, we approximated the stroke plane as horizontal and the normalized lift profile to have the same shape as the reported vertically oriented force profile (‘Normalized Lift Profile Proxy’), so that the lift generated during a wingbeat summed up to body weight for all associated species in the same way. ** and ***: these forces do not equate to lift, but we used the normalized profile as an approximation for the lift profile. * and ***: these forces do not necessarily equate to body weight when integrated over a wingbeat in hover. **: these forces do equate to body weight when integrated over a wingbeat in hover. * and ** and ***: the normalized profiles of these forces were used and either equate to or are a proxy for the lift profiles. [file elife-63107-supp5.docx]

| Paradigm Animal | Representative Group | Wingbeat Freq. [Hz] | Mass  [g] | Wing Length  [mm] | Stroke Amplitude  [°] | Normalized Lift Profile Proxy  [-] |
| --- | --- | --- | --- | --- | --- | --- |
| *Culex quinquefasciatus* | Elongated Flies | 717 | 0.0012 | 2.8 | 39 | Lift Force * |
| *Drosophila*  *hydei* | True Flies | 189 | 0.001 | 3 | 70 | Net Force *** |
| *Manduca*  *sexta* | Butterflies and Moths | 29 | 1.4 | 51 | 45 | Lift Force * |
| *Calypte*  *anna* | Hummingbirds | 44 | 4.8 | 53 | 72 | Vertical Force ** |
| *Forpus*  *coelestis* | Generalist Birds | 20 | 28 | 100 | 66 | Vertical Force ** |
